# Remimazolam as a Novel Option for Procedural Sedation: A Narrative Review

**DOI:** 10.7759/cureus.91962

**Published:** 2025-09-10

**Authors:** Hubert Ziembicki, Natalia Skrzypska, Justyna Wróblewska, Kacper Zagaja, Paulina Wasilewska, Olga Wojtczak

**Affiliations:** 1 Clinical Department of Anesthesiology, Intensive Care, and Pain Management, University Clinical Hospital, Poznań, POL; 2 Hospital Medicine, University Clinical Hospital, Poznań, POL

**Keywords:** benzodiazepines, procedural sedation, remimazolam, remimazolam clinical use, remimazolam efficacy, sedative agents comparison, short-acting sedatives

## Abstract

Procedural sedation (PS) is a widely used practice designed to relieve anxiety, induce amnesia, and improve patient comfort during diagnostic and therapeutic procedures, particularly those that may cause discomfort. The choice of sedative agent plays a critical role in determining the depth of sedation, recovery time, and risk of adverse effects. Traditionally, midazolam, a benzodiazepine (BDZ), has been the agent of choice for PS due to its rapid onset and relatively short duration of action. However, midazolam’s active metabolites can result in prolonged sedation, posing a risk of delayed recovery and increased complications. These drawbacks have sparked interest in the development of alternatives that offer a more favorable pharmacokinetic profile. Remimazolam, a novel ultra-short-acting BDZ, has emerged as a potential solution. Unlike midazolam, remimazolam has a rapid onset of action, a very short duration of effect, and no active metabolites, which significantly reduces the risk of prolonged sedation and enhances patient safety. Its unique pharmacological properties make it a promising candidate for PS, particularly in outpatient and short-duration procedures where rapid recovery is critical.

This narrative review aims to evaluate the available literature on remimazolam as an agent for PS, with a focus on its pharmacology, safety profile, and clinical efficacy. A comprehensive literature search was conducted on PubMed and Google Scholar, focusing on studies published between 2015 and 2025. The review encompasses original research articles, clinical trials, systematic reviews, and preclinical studies that compare remimazolam with other sedatives, including midazolam, propofol, and ketamine. The findings from these studies were synthesized to highlight the advantages of remimazolam, including its rapid onset, short duration, and safety profile. Emphasis was placed on clinical studies assessing its efficacy in various procedural contexts, identifying gaps in the current evidence, and suggesting directions for future research.

## Introduction and background

Procedural sedation (PS) is commonly defined as a pharmacological method of suppressing a patient’s consciousness to conduct short-period diagnostic or therapeutic procedures [1,2]. PS reduces anxiety and induces amnesia, which increases patients’ comfort before specific unpleasant diagnostic and therapeutic procedures [3]. It is widely used in hospital and ambulatory settings, with physicians educated in various fields, often outside of anesthesiology, implementing this method [4]. The European Society of Anaesthesiology and Intensive Care states that the growing availability of short-acting, relatively safe anesthetic agents has facilitated increasing interest in PS, also among patients. Over the recent years, PS has become an integral part of diagnostics and therapy in modern medicine [5].

Various pharmacologic agents are commonly used for this practice, providing different pharmacokinetic and pharmacodynamic profiles. The chosen drug determines the course of the sedation and the risk of adverse events. Therefore, the correct choice is pivotal. The sedative selected not only shapes the safety profile of the procedure but also influences recovery time, discharge readiness, and overall procedural efficiency, underscoring its practical importance beyond pharmacology alone. The ideal agent for PS should be characterized by rapid onset, short-acting time, fast recovery, appropriate titration modalities, and low risk of adverse events [5-8].

Most commonly used anesthetic agents in PS include benzodiazepines (BDZs), propofol, and ketamine. Several BDZs are utilized in PS, including diazepam, lorazepam, and midazolam. Among various BDZs, midazolam has long been considered the gold standard for PS, mainly due to its quick onset and shorter sedation period, making its pharmacological profile more favorable than other BDZs. However, it has long been known that midazolam has an active metabolite, which exhibits sedative properties and can cause prolonged sedation [9]. This unfavorable midazolam characteristic has sparked a search for a new, better BDZ for PS.

Recent developments in pharmacological fields of science have led to the synthesis of remimazolam, a novel ultra-short-acting BDZ administered intravenously (IV). Remimazolam is distinguished from other BDZs by its unique pharmacokinetics - rapid onset and ultra-short time of action. Additionally, its safety profile appears superior to other BDZs. Remimazolam, with its distinct pharmacology, positions itself as a promising anesthetic option for PS. This review aims to investigate remimazolam as a potential anesthetic agent of choice in PS.

## Review

Methods

Study Design

This narrative review aims to provide a comprehensive summary of the available literature on remimazolam as an emerging agent for PS, focusing on its pharmacological properties and potential advantages compared to other commonly used sedatives. The clinical evidence surrounding remimazolam is critically analyzed, with particular emphasis on its pharmacodynamic profile, safety, and efficacy in various procedural settings. The review highlights how remimazolam may offer distinct benefits in PS compared to traditional short-acting benzodiazepines, such as midazolam, and other sedative agents.

An extensive search was conducted across PubMed and Google Scholar databases, covering publications through 2025, with a primary focus on studies published between 2015 and 2025. Older studies were included when more recent data were insufficient or absent, ensuring a thorough overview of the existing literature on the subject. The search strategy employed Boolean combinations of the following keywords: (“remimazolam” OR “procedural sedation” OR “sedative agents” OR “short-acting benzodiazepines” OR “sedation anesthetic agents”) AND/OR (“remimazolam pharmacology” OR “sedation pharmacodynamics” OR “remimazolam safety” OR “remimazolam efficacy” OR “remimazolam sedation efficacy” OR “remimazolam versus midazolam” OR “sedative drugs for procedural sedation”).

The inclusion criteria for this review encompassed peer-reviewed original research articles, clinical studies, systematic reviews, and other relevant reviews that focused on the use of remimazolam in PS. Emphasis was placed on clinical trials comparing remimazolam with other sedatives, particularly midazolam and propofol, assessing parameters such as onset of action, duration of sedation, safety, and overall patient outcomes. In cases where clinical data were limited or unavailable, preclinical studies, pharmacokinetic data, and other relevant sources were considered to provide a broader understanding of remimazolam’s potential in this context. Exclusion criteria were applied to studies that did not focus on PS or examined remimazolam in unrelated contexts, such as ICU sedation or general anesthesia.

A meta-analysis was not included due to significant heterogeneity across the studies, including differences in patient populations, procedural types, and outcome measures. Such variability prevented meaningful statistical analysis, as pooling data would compromise the reliability of the results. Instead, the review emphasizes the identification of recurring themes, potential advantages, and notable gaps in the current body of evidence. By discussing these aspects, the review aims to guide future research directions and improve clinical practices related to the use of remimazolam in PS, ultimately informing medical professionals about the practical applications of this novel sedative agent.

Risk of Bias

As a narrative review, the lack of a structured systematic review methodology and standardized quality assessment procedures can lead to potential bias in the selection of studies. The studies included were chosen based on the authors' discretion rather than by following a set of predefined criteria. While efforts were made to ensure comprehensive coverage of relevant literature through Boolean search strategies in PubMed and Google Scholar, reliance on these databases may have resulted in the exclusion of studies published elsewhere.

Moreover, the wide variation in the included studies, such as differences in patient populations, procedural contexts, sedative agents, and outcome measures, limited the ability to directly compare results or conduct a meta-analysis. Consequently, the review focuses on synthesizing qualitative data and categorizing findings thematically, rather than evaluating quantitative data such as sedation duration or specific efficacy outcomes.

It should also be acknowledged that publication bias is a concern. Studies reporting favorable or promising outcomes are more likely to be published and cited, which could skew the available evidence in favor of positive results. Additionally, while the review prioritized literature from 2015 to 2025 to capture the most current developments, older studies, which may still hold clinical relevance, could have been underrepresented.

Finally, some degree of interpretive bias may have arisen from the subjective nature of the synthesis process and the selective emphasis on specific findings. Although the review aimed to offer an objective and balanced overview, the conclusions presented should be interpreted carefully. This review is intended to serve as a starting point for further research, rather than as a definitive clinical guide.

Pharmacology of remimazolam

Pharmacodynamics and Pharmacokinetics

Remimazolam, similarly to other BDZs, is a positive allosteric modulator of the γ-aminobutyric acid type A (GABA_A_) receptor in the central nervous system (CNS), enhancing the effect of endogenous γ-aminobutyric acid (GABA) and thereby producing CNS depression [10,11]. Remimazolam modulates four major GABA_A_ receptor subtypes (α1, α2, α3, and α5), displaying marginally higher potency toward the α1 subtype [12,13]. Remimazolam is structurally similar to midazolam; however, it can be characterized as a “soft-drug”, because in its chemical structure, similarly to remifentanil and unlike midazolam, there is a carboxylic ester group, which enables fast hydrolysis and drug inactivation [14,15]. After hydrolysis, remimazolam is transformed into its inactive metabolite, CNS 7054, which presents significantly (300 to 400 times) lower affinity for the GABA_A_ receptor [16]. Due to this property, remimazolam delivers ultra-short and dose-dependent sedation, which facilitates precise titration and low residual sedation risk [13]. Schüttler et al. (2020) confirmed in clinical trials that remimazolam is characterized by lower interindividual variability compared to midazolam, which makes it more easily controllable and more predictable [17].

Adverse Effects and Tolerance

Hypotension has been reported as an adverse effect of remimazolam; however, it still displays favorable hemodynamic stability compared to other IV anesthetic agents [16]. It exhibits weak inhibition of the hERG tail current, with 25% and 50% inhibitory concentration values estimated at 62 μM and 207 μM, respectively, indicating a low propensity for hERG-mediated cardiac effects [12]. IV anesthetic agents, besides ketamine, induce a decrease in systemic vascular resistance and cause cardiac depression by reducing its contractility. Because of structural similarities, remimazolam displays a midazolam-like effect on BP, presenting a more favorable hemodynamic profile than other IV anesthetic agents.

There is limited clinical data regarding tolerance to remimazolam. Io et al. (2021) examined tolerance to different BDZs in a pig model over 28 days. They assessed tolerance by comparing the required dose upshift to maintain a constant level of sedation. The dose increase for remimazolam was significantly lower than that of midazolam. This suggests tolerance to remimazolam might be less than to midazolam in long-term administration in humans; however, more clinical data is needed [18].

Reversal of Action

The possibility of reversing the action is an important advantage when choosing a sedative agent for PS [5]. It allows for faster patient recovery after sedation and offers a safety backup should an anesthetic agent be overdosed. Flumazenil is a selective antagonist of the GABA_A_ receptor, approved and clinically used for reversing BDZ action after administration [19]. The use of flumazenil as a reversal agent for remimazolam has been widely reported in both clinical and pre-clinical studies [13,20]. Chen et al. (2020) reported that flumazenil provides quick reversal of action after remimazolam administration and enhances hemodynamic recovery after sedation due to its cardiostimulating properties [20].

Currently, routine use of flumazenil to reverse midazolam effects in PS is considered poor clinical practice and discouraged because of the possibility of re-sedation [21]. Sneyd et al. (2020) suggested that the shorter effect duration of remimazolam causes lower re-sedation risk compared to midazolam and might allow for better flumazenil incorporation into routine end-of-sedation schemes [22]. The consensus is that flumazenil reversal of remimazolam does not pose significant re-sedation risks [23]. However, reports about isolated cases of re-sedation are emerging [24]. More extensive clinical research is needed to assess the risks of re-sedation and potential incorporation of flumazenil in routine end-of-sedation protocols in remimazolam-induced PS.

Remimazolam in comparison with other sedative agents

Remimazolam remains less popular than other sedative agents used in PS. It seemingly exhibits many beneficial properties, with numerous advantages over other commonly used agents. A summary of a comparison between commonly used PS sedative agents and remimazolam is provided in Table 1.

**Table 1:** Comparison of pharmacological properties of chosen sedative agents used in procedural sedation IV: intravenous; CYP3A4: cytochrome P450 3A4; CYP450: cytochrome P450

Sedative agent	Onset of action after IV administration	Duration of action	Metabolism	Active metabolites	Cardiovascular effects	Respiratory effects	Rapid titration	References
Remimazolam	1–2 min	5–20 min	Tissue esterases (organ-independent)	No	Minimal depression	Minimal depression	Possible	[8,13,25,26]
Midazolam	1–2 min	15–80 min	Hepatic (CYP3A4)	Yes	Mild depression	Dose-dependent depression	Not possible	[5,27,28]
Propofol	<1 min	4–8 min	Hepatic (CYP450, glucuronidation)	No	Depression	Dose-dependent depression	Possible	[28,29,30,31]
Ketamine	<1 min	10–15 min	Hepatic (CYP450)	Yes	Stimulation	Preserved drive, rare depression	Not possible	[28,32]

Remimazolam Versus Propofol

Propofol is widely utilized as a sedative agent in PS [29]. Propofol is a short-acting positive modulator of the GABA_A_ receptor administered IV. Like remimazolam, it causes rapid loss of consciousness. It also exhibits amnestic effects, similar to those of remimazolam and other BDZs [30]. Propofol is also considered to be a relatively safe sedative agent, with fewer adverse effects compared to many other anesthetic agents [33].

 A significant depressive effect on the cardiovascular system caused by propofol remains a major disadvantage when used in PS [34]. This limits its use in hypotensive patients, elderly patients, and patients with concurrent heart disease. Propofol inhibits L-type calcium channels in cardiac myocytes and vascular smooth muscle, clinically manifesting as bradycardia, hypotension, and negative inotropy [35,36]. Moreover, propofol can mobilize calcium ions from the endoplasmic reticulum (ER) and elevate calcium-mediated depressive effects on the cardiovascular system further [37]. In contrast, remimazolam, like other BDZs, has minimal effects on the cardiovascular system, making it a more favorable choice in terms of hemodynamic stability. Unlike propofol, it does not significantly affect L-type calcium channels.

Urabe et al. (2022) investigated the elevation of intracellular calcium after remimazolam administration, proving it increases calcium levels through G-protein-coupled inositol 1,4,5-triphosphate receptors. They stress that this effect is reversible, unlike after propofol administration, in which case this effect is mediated by different pathways and irreversible [38]. This significantly smaller impact on calcium homeostasis is a clear advantage, as it reduces the risk of adverse cardiovascular effects such as excessive hypotension and cardiac depression. Consequently, remimazolam offers a more controlled and predictable action on the cardiovascular system, enhancing its safety profile, particularly in patients with sensitive circulatory conditions.

Remimazolam offers a pharmacological profile similar to that of propofol, with both agents acting on GABA_A_ receptors to induce sedation and anesthesia. Both drugs are short-acting and have a rapid onset of action, making them suitable for PS. However, remimazolam has the advantage of a more favorable cardiovascular profile, as it exerts minimal effects on blood pressure and heart rate compared to propofol, which is known for its significant depressive effects on the cardiovascular system [16]. Chang et al. (2023) reported that patients under PS sedated with remimazolam had a significantly lower risk of bradycardia, hypotension, and respiratory depression than those sedated with propofol [39]. Clinically, this suggests a particular advantage in patients who are vulnerable to hemodynamic or respiratory compromise. In such cases, the combination of cardiovascular stability with rapid and predictable recovery may reduce the need for rescue interventions and enhance overall safety, while still allowing adaptation to different procedural contexts.

These advantages may be especially relevant in outpatient settings, where rapid turnover and early discharge are priorities. They also carry significance for populations in whom even modest instability can have serious consequences, such as individuals with pre-existing cardiac or respiratory disease. Additionally, remimazolam benefits from the availability of flumazenil as a specific reversal agent, allowing for the rapid reversal of sedation in case of over-sedation. In contrast, propofol lacks a dedicated reversal agent, which can complicate the management of overdose or prolonged sedation, further highlighting the safety advantages of remimazolam.

Available studies focus on the favorable cardiovascular profile of remimazolam compared to propofol [39,40]. More extensive research is needed to fully compare these two agents in clinical settings and to evaluate the long-term safety of remimazolam in diverse, larger patient populations.

Remimazolam Versus Ketamine

Ketamine is a frequently used sedative agent in PS, characterized by a relatively quick onset and short duration of action. It can be administered IV or intramuscularly (IM) and is an antagonist of the N-methyl-D-aspartate (NMDA) receptor and a partial agonist of μ-opioid receptor (MOR), providing dissociative anesthetic and analgesic effects [32]. These unique properties allow for consistent sedation and analgesia even in painful procedures, without the need for additional analgesics. Ketamine also displays beneficial cardiovascular effects. Unlike most of the sedatives, it stimulates the sympathetic system, resulting in increased stroke volume, contractility of the heart, and BP.

While ketamine induces dissociative anesthesia, characterized by a profound separation of sensory input and consciousness, it can also lead to undesirable side effects. Strayer et al. (2008) reported a significantly high incidence of an undesirable side effect of ketamine in PS, known as emergence phenomena, which is an unfavorable occurrence [41]. This phenomenon can lead to confusion, agitation, and hallucinations as patients regain consciousness, making it particularly problematic in settings where rapid recovery and patient comfort are essential. These effects make ketamine less favorable in certain clinical settings, particularly in patients with pre-existing neurological or psychiatric conditions. In contrast, remimazolam provides a more predictable and controlled sedative effect, acting via GABA_A_ receptors to induce sedation without the dissociative state seen with ketamine.

Although the cardiovascular profile of ketamine may be beneficial for certain patient groups, for many patients, including those with a compromised cardiovascular system, remimazolam offers a more favorable option [42]. Kim et al. (2022) reported that remimazolam does not exert a significant impact on the cardiovascular system, making it a safer choice for patients with pre-existing cardiovascular conditions, such as hypertension or heart disease [16]. Additionally, the safety profile of remimazolam is enhanced by its reversibility with flumazenil. This contrasts with ketamine, which lacks a specific reversal agent and can present challenges in managing over-sedation or adverse effects. In terms of efficacy, remimazolam offers a rapid onset and short duration, making it highly suitable for short procedures, while providing more predictable and manageable sedation compared to the variable effects of ketamine.

Remimazolam offers several advantages over ketamine in PS. Unlike ketamine, which is associated with dissociative effects and emergence phenomena, remimazolam provides more predictable and controlled sedation [40]. Its minimal impact on the cardiovascular system makes it a safer option for patients with underlying cardiovascular conditions, whereas ketamine may pose risks in such populations [23,42]. Furthermore, remimazolam's reversibility with flumazenil enhances its safety profile, positioning it as a more favorable choice for PS. 

Despite clear advantages of remimazolam over ketamine, there is virtually no clinical data directly comparing both drugs in PS. Further studies are needed to fully evaluate their relative safety, efficacy, and hemodynamic profiles in various patient populations. Direct head-to-head comparisons will be essential to establish the optimal anesthetic choice for different clinical settings.

*Remimazolam Versus Midazolam*

Midazolam is a widely used BDZ sedative agent utilized in PS. It exhibits a relatively fast onset; however, its duration of action is significantly longer than that of propofol and remimazolam [5,43]. Similarly to remimazolam, it is a positive allosteric modulator of the GABA_A_ receptors, inducing sedative, anxiolytic, and amnestic effects. Midazolam is typically administered IV, and its primary use is for PS in various medical procedures. 

Midazolam is primarily metabolized in the liver by cytochrome P450 3A4 (CYP3A4), undergoing hydroxylation to form active metabolites such as 1'-hydroxymidazolam [27]. In patients with liver disease, midazolam metabolism is problematic due to impaired hepatic function. Hasegawa et al. (2017) in their experimental rat models proved that hepatic impairment or CYP inhibition can increase the plasma concentrations of midazolam metabolites, potentially leading to unexpected pharmacodynamic effects and toxicity [44]. In contrast, remimazolam is rapidly hydrolyzed by plasma and hepatic esterases into an inactive metabolite, leading to a significantly shorter duration of action and a more predictable recovery time. This faster metabolism makes remimazolam a safer option, especially in patients with liver dysfunction, as it reduces the risk of prolonged sedation, associated complications, and toxicity.

Remimazolam, a newer benzodiazepine, shares the same GABA_A_ receptor-mediated sedative action but offers a more favorable cardiovascular profile. Unlike midazolam, remimazolam exerts minimal effects on blood pressure and heart rate, making it safer for patients with underlying cardiovascular concerns, such as hypertension or heart disease. Tang et al. (2022) reported that remimazolam is associated with a lower incidence of hypotension and bradycardia compared to midazolam [40]. This favorable cardiovascular effect makes it a better choice for patients with preexisting heart disease.

AbuJwaid et al. (2025) reported that remimazolam shortened induction time by approximately three minutes and hastened recovery time compared to midazolam in PS for bronchoscopy. Additionally, they reported that patients who received remimazolam less often required additional rescue anesthesia compared to midazolam [45]. Another clinical study by Pambianco et al. (2016) examined patients undergoing PS for colonoscopy. Remimazolam provided significantly higher sedation success rates than midazolam, with 92% and 75% respectively [46]. Other clinical trials by Zhu et al. (2021) and Pastis et al. (2019) provide similar conclusions, advocating for higher sedation efficacy of remimazolam versus midazolam [47,48]. Midazolam and remimazolam provide a reversal possibility with flumazenil; however, remimazolam offers a significantly lower risk of re-sedation [23]. This allows for greater patient safety in potential action reversal when remimazolam is administered.

Numerous clinical studies and reviews have directly compared midazolam and remimazolam in PS. These studies consistently demonstrate that remimazolam offers superior efficacy, faster induction and recovery times, and a more favorable cardiovascular profile. Unlike midazolam, which can cause hypotension and bradycardia, remimazolam has minimal effects on blood pressure and heart rate, making it safer for patients with underlying cardiovascular concerns. Clinical studies have demonstrated that remimazolam not only provides faster induction and recovery times but also achieves higher sedation success rates compared to midazolam. Its greater efficacy and lower risk of re-sedation further enhance its safety and reliability in PS. These characteristics make remimazolam a preferable choice, particularly in patients with pre-existing cardiovascular conditions.

Challenges in the clinical application of remimazolam

*Lack of Clinical Data*

Remimazolam, although a promising agent in PS, remains relatively new compared to established sedatives such as midazolam and propofol. While initial studies and clinical trials have shown its efficacy and safety, its long-term effects and performance across a broad range of clinical settings are still being explored. The limited experience with remimazolam may pose a challenge in widespread adoption, as healthcare professionals may be less familiar with its pharmacodynamics, potential adverse reactions, and the management of sedation in complex cases. As more data accumulates from diverse patient populations, clinical practice guidelines will likely evolve to provide clearer recommendations for their use.

Cost and Accessibility

One of the practical challenges in the broader use of remimazolam is its cost relative to older sedative agents like midazolam. Hassan et al. (2024) reported that midazolam remains the most cost-effective sedative in PS in dentistry [49]. This could pose barriers to widespread adoption of remimazolam, particularly in resource-limited settings or for procedures where cost-effectiveness is a significant consideration. While the availability of remimazolam becomes more widespread, it may be restricted in certain regions or institutions, which may limit its use in clinical practice, despite its advantages in safety and efficacy [50].

*Lack of Consensus on Dosing*

Despite the favorable pharmacokinetics and safety profile of remimazolam, optimal dosing regimens for various PS scenarios remain under investigation [51,52]. Variability in patient response, especially among those with comorbid conditions such as renal or hepatic impairment, may complicate the determination of ideal dosing protocols. Furthermore, the limited experience in different clinical contexts means that healthcare providers may need to tailor doses individually, which could lead to inconsistencies in sedation depth and recovery time. Ongoing research is needed to establish more definitive guidelines for dosing, especially in diverse patient populations, to ensure both efficacy and safety in clinical practice.

Key findings

Remimazolam offers notable overall advantages over midazolam, ketamine, and propofol, including a more favorable cardiovascular profile with minimal effects on blood pressure and heart rate. It provides faster induction and recovery times, as well as higher sedation success rates. Despite these benefits, several challenges remain, such as limited clinical experience, cost, and the need for further research on dosing protocols.

## Conclusions

Remimazolam represents a significant advancement in PS, offering a range of benefits over traditional sedatives like midazolam, propofol, and ketamine. Its ultra-short duration of action and the absence of active metabolites make it a promising choice for procedures requiring rapid recovery. Unlike midazolam, which may cause prolonged sedation due to its active metabolites, remimazolam offers more predictable sedation and a lower risk of complications, particularly in patients with cardiovascular concerns. The minimal effects on blood pressure and heart rate distinguish it from agents like propofol, which can induce hypotension and bradycardia. Remimazolam is preferable to ketamine in PS due to its more predictable and controlled sedative effect and minimal cardiovascular impact. Additionally, its reversibility with flumazenil, coupled with its favorable cardiovascular profile, enhances patient safety, making it a preferable option for patients with pre-existing cardiovascular conditions. However, the current body of evidence is limited by the novelty of the drug, and further research is needed to refine dosing protocols and assess long-term outcomes. Challenges such as cost and the need for widespread clinical adoption also remain. Overall, while remimazolam offers promising advantages, its integration into clinical practice will require overcoming these barriers, and further studies will be crucial to fully establish its role in PS.
